# Interlayer Sliding Phonon Drives Phase Transition in the Ph-BTBT-10 Organic Semiconductor

**DOI:** 10.1021/acs.chemmater.3c00209

**Published:** 2023-07-20

**Authors:** Elena Ferrari, Lorenzo Pandolfi, Guillaume Schweicher, Yves Geerts, Tommaso Salzillo, Matteo Masino, Elisabetta Venuti

**Affiliations:** † Dipartimento di Scienze Chimiche, della Vita e della Sostenibilità Ambientale & INSTM-UdR Parma, Parco Area delle Scienze, 17/A, Parma 43124, Italy; ‡ IMEM-CNR, Parco Area delle Scienze, 37/A, Parma 43124, Italy; ¶ Dipartimento di Chimica Industriale “Toso Montanari” & INSTM-UdR Bologna, Viale del Risorgimento 4, Bologna 40136, Italy; § Laboratoire de Chimie des Polymères Faculté des Sciences, Université Libre de Bruxelles (ULB), Boulevard du Triomphe, CP 206/01, Brussels 1050, Belgium; ∥ International Solvay Institutes of Physics and Chemistry Université Libre de Bruxelles (ULB), Boulevard du Triomphe, CP 206/01, Brussels 1050, Belgium

## Abstract

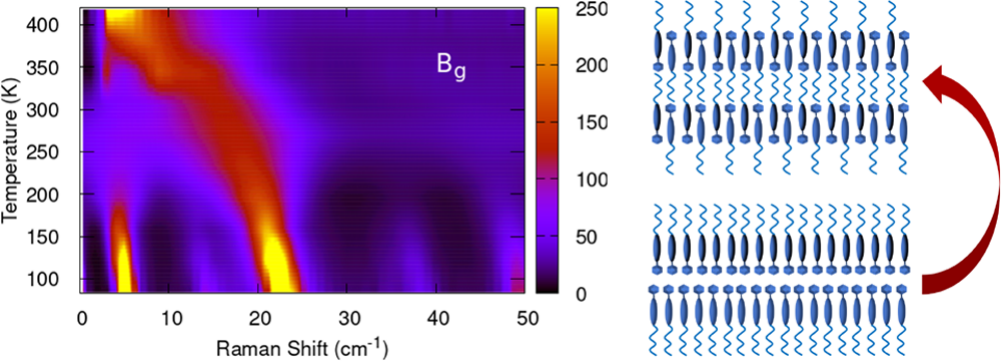

In the field of organic electronics, the semiconductor 7-decyl-2-phenyl[1]benzothieno[3,2-*b*][1]benzothiophene (Ph-BTBT-10) has become a benchmark due to its high charge mobility and chemical stability in thin film devices. Its phase diagram is characterized by a crystal phase with a bilayer structure that at high temperature transforms into a Smectic E liquid crystal with monolayer structure. As the charge transport properties appear to depend on the phase present in the thin film, the transition has been the subject of structural and computational studies. Here such a process has been investigated by polarized low frequency Raman spectroscopy, selectively probing the intermolecular dynamics of the two phases. The spectroscopic observations demonstrate the key role played by a displacive component of the transition, with the interpenetration of the crystal bilayers driven by lattice phonon mode softening followed by the intralayer rearrangement of the molecule rigid cores into the herringbone motif of the liquid crystal. The mechanism can be related to the effectiveness of thermal annealing to restore the crystal phase in films.

## Introduction

Displacive phase transitions are structural transformations, common for inorganic periodic systems, that only require small collective displacements of individual constituents of the crystal. For instance, the phase transition at 105 K in SrTiO_3_ has become one of the archetype examples of the class alongside those of systems such as quartz and ferroelectric perovskites. In SrTiO_3_ the tetragonal to cubic phase transformation is accompanied by the softening of a vibrational mode measured by both Raman^1^ and neutron scatterings.^2^ In fact, following the soft-mode theory of displacive phase transitions, the transition occurs as the result of some phonon frequency going to zero at a critical temperature.^3,4^

Notably, displacive transitions are not commonly encountered in organic molecular crystals, with the exception of the Peierls type neutral–ionic transitions typical of charge transfer complexes at low temperature or high pressure, which lead to the dimerization of the donor–acceptor molecules.^5−7^ However, concerted molecular displacements associated with a specific normal mode which result in a new phase have also been invoked for the molecular crystal DL-norleucine, which undergoes entire bilayer shifts during a displacive transformation.^8^

In the field of organic electronics, the compound 7-decyl-2-phenyl[1]benzothieno[3,2-*b*][1]benzothiophene (Ph-BTBT-10) has become a benchmark material because of its high charge mobility and chemical stability even in thin films,^9,10^ unlike pentacene and rubrene, the most studied systems in the past.^11−13^

**Figure 1:**
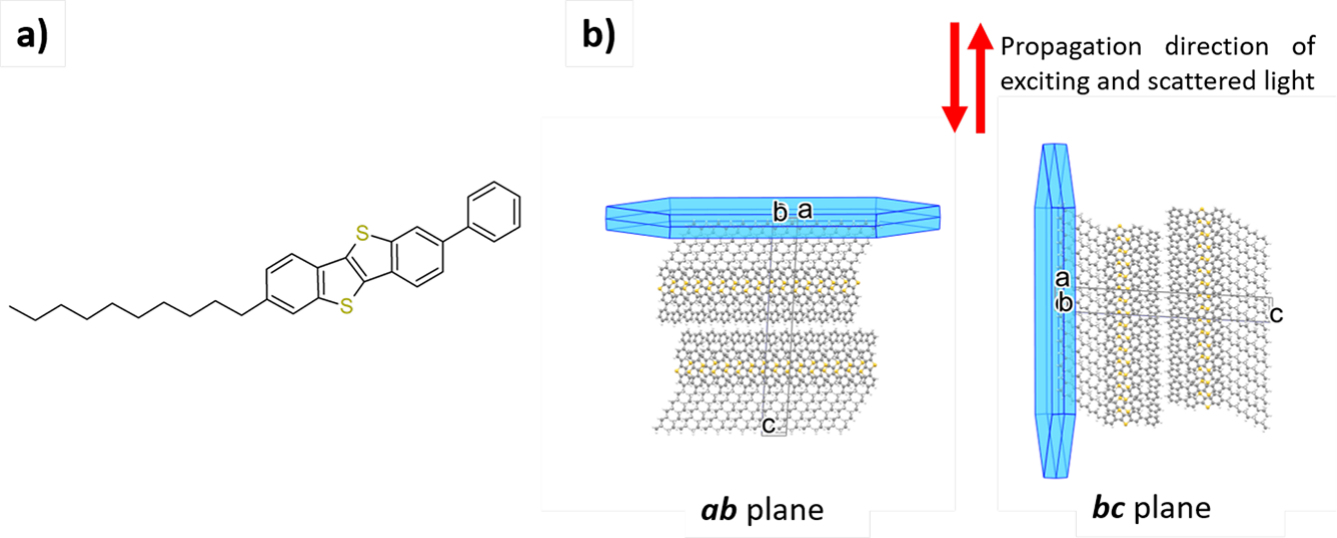
(a) Ph-BTBT-10 molecular structure. (b) Two crystal orientations probed. The red arrows indicate the propagation direction of the light.

In Ph-BTBT-10 the rigid BTBT core is functionalized with a phenyl group and a flexible decyl chain (Figure 1a), in a structure designed to achieve both good solubility and ordered liquid crystal phases, which are precursors to the formation of uniform crystalline thin films with increased 2-D mobility.^9,10^

Due to the asymmetric substitution, Ph-BTBT-10 crystallizes in a bilayer structure, with a monoclinic unit cell where the *ab* plane is parallel to the layers and the long molecular axis is nearly parallel to the *c* axis (Figures 1b, S1, and S2). The strong interactions of the BTBT cores result in their herringbone arrangement and segregation from the decyl chains.^14^

Ph-BTBT-10 is reported to undergo three first order phase transitions on heating: (i) Crystal to SmE at 150 °C; (ii) SmE to SmA at 215 °C; and finally (iii) SmA to isotropic liquid at 225 °C.^15^ In the first one the structure changes from bilayer head-to-head to monolayer head-to-tail, with two adjacent antiparallel molecules in the unit cell.^16,17^ Since charge transport properties in thin films appear to be regulated by transformations between crystalline and SmE phases,^15,17−20^ a deeper understanding of the underlying processes is desirable.

In this work, we report on a low frequency Raman study aimed at clarifying the mechanism of the crystal to smectic E phase transition of Ph-BTBT-10. In fact, low frequency Raman spectroscopy is highly sensitive to the 3D arrangement of the crystal state by probing the intermolecular dynamics and therefore detecting the patterns of interaction. Polarized Raman measurements on oriented single crystals were used to probe the crystal planes *ab*, parallel to the molecular layers, and *bc*, perpendicular to them, allowing for the qualitative description of the lattice modes in terms of the crystal interactions patterns. Measurements as a function of the temperature revealed the existence of mode softening, providing direct information about the displacive nature of the transition. However, the spectral features also show evidence of a discontinuity, demonstrating that the overall transformation process involves a two step mechanism.

## Results and discussion

### Room Temperature Raman Spectra

The Ph-BTBT-10 crystals display an elongated platelet morphology, with the two in-plane symmetry axes coinciding with the extinction directions under crossed polarized light. The observed morphology completely agrees with the prediction of the BFDH model (Bravais, Friedel, Donnay, and Harker)^21^ for the monoclinic *P*2_1_/*a* structure (Figure S2), allowing for the assignment of the longer and shorter in-plane axes to the *a* and *b* crystallographic directions, respectively. Both of these directions are parallel to the molecular layers and nearly perpendicular to the long Ph-BTBT-10 molecular axis. The observed morphology originates from a faster growth along *a* and *b*, driven by the strong in-plane interactions between the aromatic cores.^14^

In the vibrational analysis of a molecular crystal, it is customary to distinguish between inter- and intramolecular modes on the basis of their different force constants, as the former depend on the weak vdW interactions and the latter on those of the chemical bonds. In the case of the flexible Ph-BTBT-10 molecule, such a distinction is made difficult by the occurrence of torsional degrees of freedom of low energy. However, we can assume that in the wavenumber range below 120 cm^–1^ most modes have predominant intermolecular character, and thus correspond to librations and translations of a (nearly) rigid molecule.

In the *P*2_1_/*a* space group, Ph-BTBT-10 Raman active modes are either of *A*_*g*_ or *B*_*g*_ symmetry: the former can be detected in the *aa*, *bb*, *cc*, or *ac* configurations of polarization, while the latter are observed in *ab* and *bc* cross-polarization. The two letters are currently adopted to label the polarized spectra and indicate the polarization directions of the exciting and scattered light, respectively.^22^

**Figure 2:**
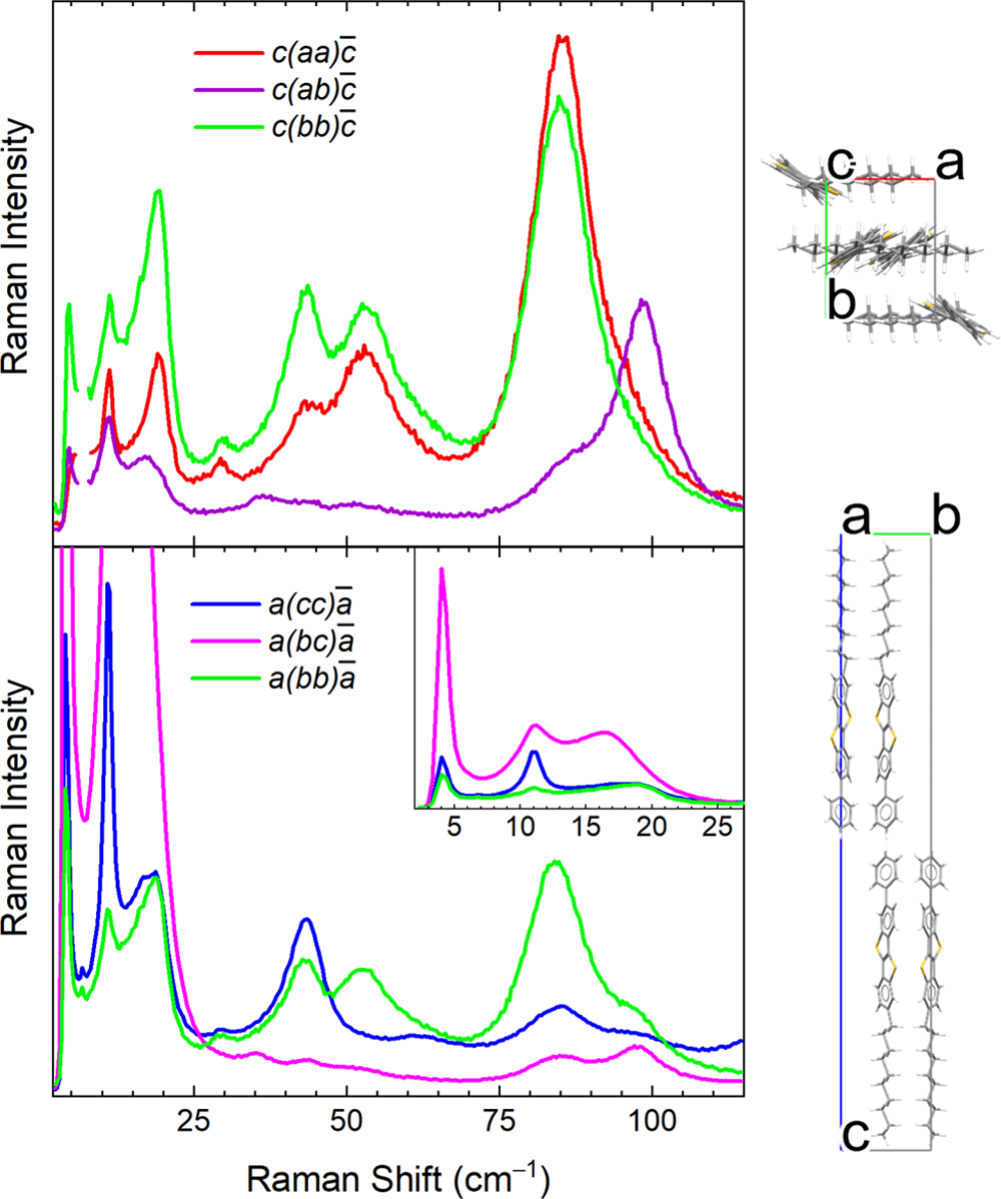
Polarized low frequency Raman spectra of crystalline Ph-BTBT-10, measured on the *ab* (upper panel) and *bc* crystal planes (lower panel). The unit cell viewed perpendicular to the corresponding planes is shown on the right side of each panel. The two letters inside parentheses are used to label the polarization of the spectra indicate the polarization direction of the exciting and scattered light, respectively. The two letters outside parentheses indicate the corresponding light propagation and scattering directions, which are perpendicular to the investigated crystal planes. The 4–25 cm^–1^ spectral range is shown in the inset with a high intensity scale for clarity. In some spectra, a narrow plasma line from the laser at 7 cm^–1^ has been removed.

In Figure 2, we report the polarized Raman spectra collected from the *ab* and *bc* crystal faces. All the in-plane polarized spectra of the crystal (i.e., *aa*, *ab*, and *bb*) show medium intensity bands around 90 cm^–1^ whereas in the out-of plane polarizations, i.e., *cc* and *bc*, very strong bands appear below 20 cm^–1^ (Table 1). As can be seen from the figure, the *aa* and *bb* spectra share the same *A*_*g*_ bands, with only small differences in the relative intensities, while modes with *B*_*g*_ symmetry can be identified in the *ab* spectrum. Interestingly, the *bc* spectrum, which probes the scattering from the corresponding crystal plane, is characterized by a huge intensity increase of the very low frequency *B*_*g*_ band by nearly an order of magnitude with respect to the *ab* plane.

**Table 1:** Vibrational Frequencies of the Ph-BTBT-10 Crystal in the Low Energy Range at 295 K^a^

Mode Frequency (cm^–1^)	Band Polarization	Mode Symmetry
4 vs	*bc*	*B*_*g*_
4 s	*cc*	*A*_*g*_
11 s	*bc*	*B*_*g*_
11 s	*cc*	*A*_*g*_
16.5 s	*bc*, *ab*	*B*_*g*_
19 m	*cc*, *bb*, *aa*	*A*_*g*_
29 w	*aa*, *bb*, *cc*	*A*_*g*_
35 w	*bc*, *ab*	*B*_*g*_
43 m	*cc*, *bb*	*A*_*g*_
53 m	*aa*, *bb*	*A*_*g*_
62 w	*cc*	*A*_*g*_
84 m	*aa*, *bb*	*A*_*g*_
95 m	*ab*, *bc*	*B*_*g*_

a The letters s (strong), m (medium), and w (weak) refer to the relative intensities of the bands. The two letters used to label the band polarization indicate the polarization direction of the exciting and scattered light, respectively.

Due to the strong anisotropy of the Ph-BTBT-10 crystalline arrangement, the modes polarized in the *ab* plane must correspond to in-plane translations or rotations about the long axes of the molecules. The out-of-plane polarized modes must instead involve translations along the long molecular axis. Such assumptions are supported by the results of the DFT simulation of the Raman spectra for the similar system C_8_O-BTBT-OC_8_.^23^ The assignment is further confirmed by the comparison between the polarized spectra of Ph-BTBT-10 and unsubstituted BTBT, which also displays a layered packing (Figure S3). Thus, in-plane polarized spectra mainly reflect intralayer molecular packing interactions, whereas the out-of-plane polarized spectra probe interlayer interactions. Accordingly, the lower frequencies of the interlayer polarized lattice phonons result from the weaker interactions between molecules belonging to adjacent layers, in agreement with the thin platelet morphology displayed by the crystal.

### Toward the Transition: The Soft Phonons

**Figure 3:**
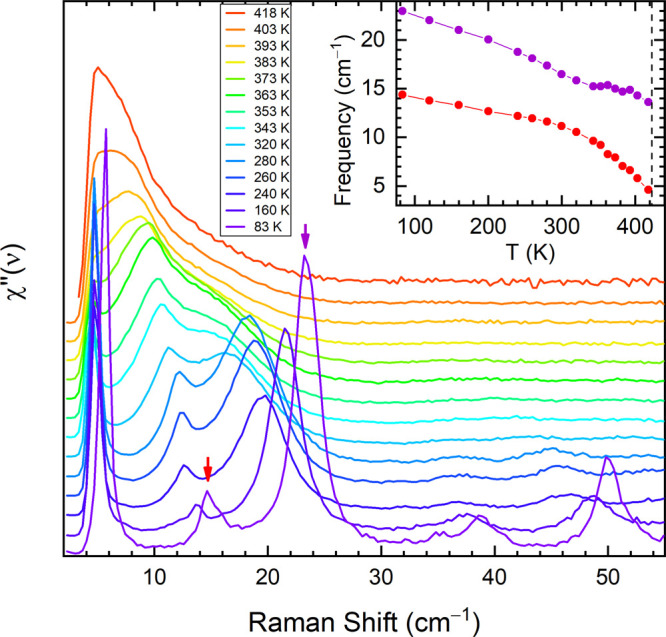
Ph-BTBT-10 low frequency Raman spectra with *bc* polarization recorded on heating from 83 to 418 K, i.e., on approaching the Crystal to SmE transition. The raw data were converted into the imaginary part of the dynamic susceptibility, *χ″* (see ref (25)). The spectra are upshifted for clarity. Inset: frequency shifts vs temperature for the two lattice modes indicated by the purple and red arrows in the main graph. The dashed line in the inset marks the transition temperature.

The soft behavior of two lattice phonons on approaching the Crystal to SmE phase transition becomes evident in the temperature evolution of the *bc* polarized spectra, as shown in Figure 3. As clearly seen in the figure, the *B*_*g*_ band centered at 23 cm^–1^ at 83 K shifts to lower energy and broadens significantly on increasing temperature. Around 300 K it closes in on the band at 12 cm^–1^, the two bands overlap, and the spectral weight is redistributed between them. Near a phase transition, the potential energy surface is expected to become strongly anharmonic, leading to the mixing of normal modes having the same symmetry and similar frequencies. Here such an effect becomes visible above 300 K, when the corresponding *B*_*g*_ modes with a large projection along the *c*-axis get strongly mixed and the soft behavior is transferred to the lower energy band, which moves toward zero frequency, while that at higher energy it gradually loses intensity and turns into a broad shoulder (see inset Figure 3).

In the same spectrum, the strong narrow band at 5 cm^–1^ is no longer detectable above 320 K, as it falls below the wavenumber detection limit, and its behavior with temperature cannot be investigated any further. For this reason, we cannot exclude a priori that the lowest frequency mode also plays a role in the transition. However, its temperature evolution in the available range, i.e., from 83 to 320 K, is characterized by the absence of sizable broadening and by minimal red-shift, suggesting a very little interaction or mixing with the other *B*_*g*_ soft phonon modes. Since the band is visible in both *bc* and *cc* polarizations, it could be assigned to an intramolecular chain mode as such low frequency phonons have been predicted in alkylated BTBT derivatives.^24^

**Figure 4:**
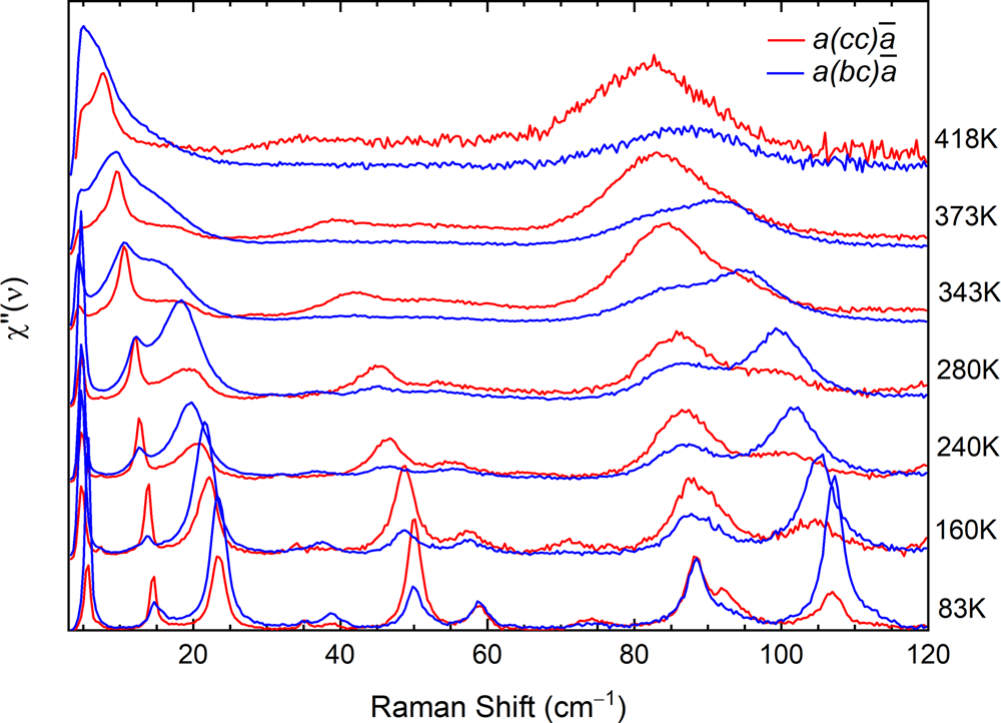
Comparison between the *bc* and *cc* polarized spectra on increasing temperature. *A*_*g*_ bands appear in the *cc* polarization, while *B*_*g*_ bands occur in the *bc* polarized spectra. The raw data were converted into the imaginary part of the dynamic susceptibility, χ*″* (see ref (25) and the main text). The spectra are upshifted for clarity.

Unlike the low frequency *B*_*g*_ phonons, *A*_*g*_ phonons display with temperature an expected typical trend, as can be seen by comparing the *cc* and the *bc* polarized spectra (see Figure 4). In particular, the *A*_*g*_ lowest frequency bands, overlapping at 83 K the *B*_*g*_ bands with soft behavior, never shift to zero frequency on increasing temperature, as shown by the plot of the mode frequencies vs temperature (Figure S5). In addition, they are narrower than their *B*_*g*_ counterparts at all temperatures. These characteristics are shared by the high frequency phonons detected in the in-plane polarized spectra, which do not display any effect that anticipates the transition (Figure S4).

### The SmE Phase

**Figure 5:**
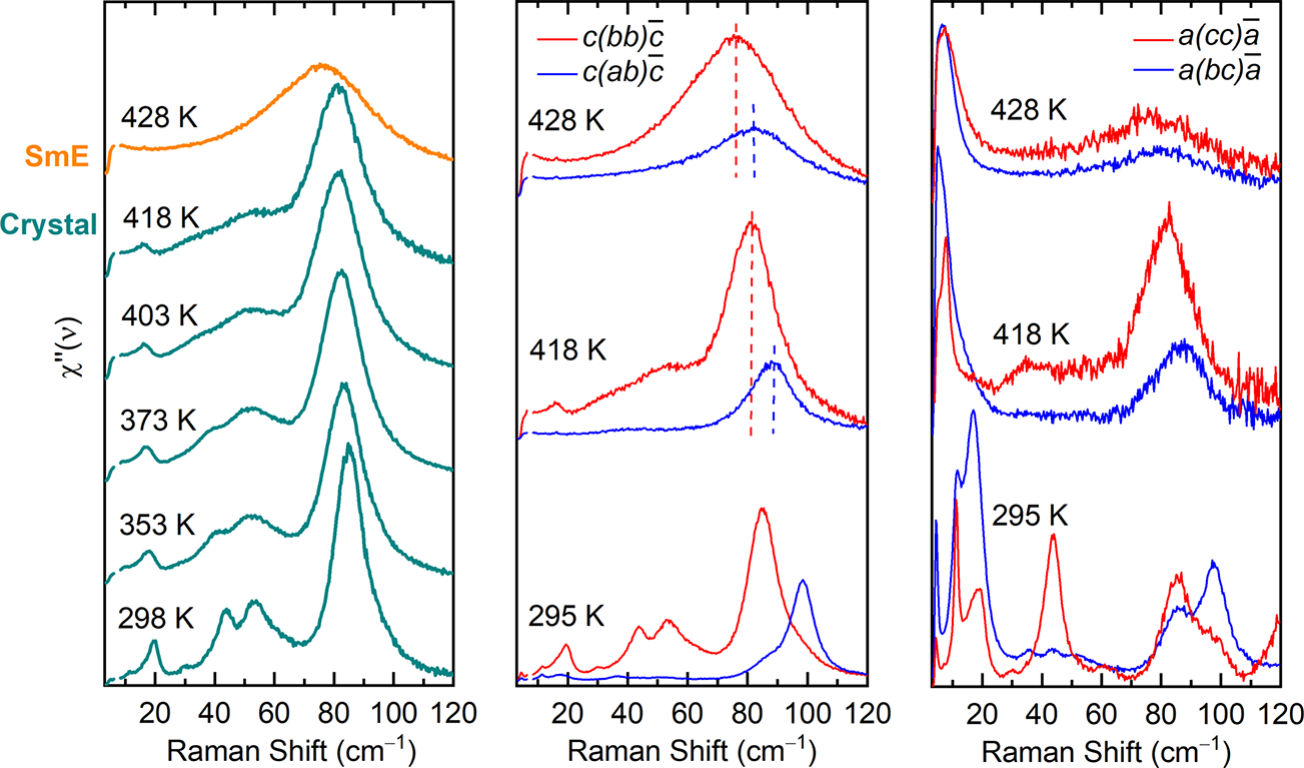
Raman spectra with *bb* polarization recorded on heating from room temperature to the Crystal to SmE transition. The green and orange lines correspond to the crystal and SmE phases, respectively (left panel). Polarized Raman spectra were recorded on *ab* (middle panel) and *bc* planes (right panel). The vertical dashed lines in the middle panel correspond to the peaks at 418 K (Crystal) and 428 K (SmE), in the parallel (red color) and crossed polarizations (blue color). The raw data were converted into the imaginary part of the dynamic susceptibility, χ*″* (see ref (25) and the main text). The spectra are upshifted for clarity.

The final occurrence of the LC SmE phase is signaled by the sudden replacement of the *bb* polarized bands of the crystal Raman spectrum with a single broad one around 70 cm^–1^ (Figure 5, left panel). The *aa* (not shown here) and *bb* spectra become fully superimposable in the new phase, while the *ab* polarized one behaves similarly but does not overlap with the other two because, like in the crystal phase, the band displays a maximum at a higher frequency (Figure 5, middle panel). In addition, the spectra of the SmE phase in the *bc* and *cc* polarizations (Figure 5, right panel) are characterized by the appearance of a strong new peak around 6 cm^–1^, which is absent in all of the in-plane polarized spectra. The complete interlayer polarization of this band suggests that it is a lattice phonon with a strong translational component along the new phase *c* axis rather than one of its intramolecular modes. In fact, the occurrence of low frequency pseudolattice vibrations have been reported in smectic phases containing antiparallel molecules^25,26^ and therefore the presence of such band in SmE Ph-BTBT-10 is consistent with its well assessed monolayer structure.

The features of the SmE low frequency spectra convey information about its organization and symmetry. Overall, for instance, the relative intensity patterns of the SmE in-plane and out-of-plane polarized scatterings are the same as in the crystal phase, demonstrating that the orientation of the layer structure is maintained in the transition. In addition, the observation that *bb* (*aa*) and *ab* polarized spectra are distinguishable is a clear indication of the presence of *ab* in-plane order. Indeed, in-plane orientational and positional orders are features characterizing the SmE phases.

### The Transition Mechanism

The bulk of spectroscopic information collected at the onset of the bilayer to monolayer phase transition must now be linked to its preparatory stage, where the experiments have detected the intervention of the softening involving crystal modes of *B*_*g*_ symmetry strongly out-of-plane polarized. More properly, the mode softening would be better described as an effective vibration, resulting from the combination of lattice phonons, as suggested by the strong anharmonicity characterizing the system dynamics on approaching the transition.

**Figure 6:**
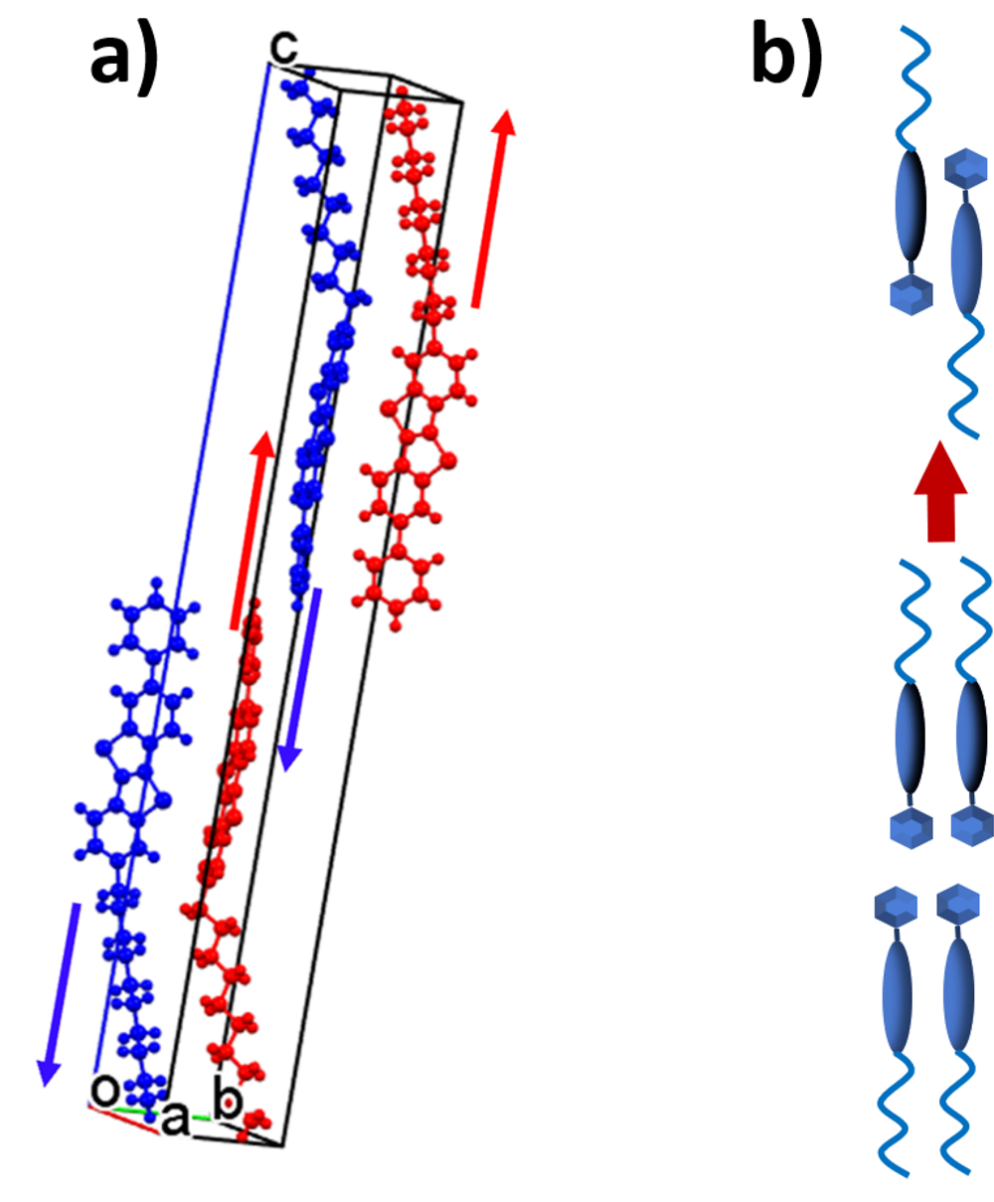
(a) Molecular motions in the lattice vibrations of *B*_*g*_ symmetry undergoing the softening on approaching the phase transition; (b) schematic representation of the molecular reorganization of the bilayer into the monolayer following the condensation of the soft mode.

In attempting the qualitative description of the responsible vibration, it must be remembered that its Raman activity implies a motion at *k* = 0, i.e., where all unit cells move in phase. An intuitive representation depicts this motion as comprehending the counter translation along the crystal *c* axis of pairs of adjacent molecules belonging to the same layer, and such a condition is satisfied by a lattice phonon of *B*_*g*_ symmetry in which two opposite layers in the unit cell move out of phase, following the scheme of Figure 6a. In fact, the interpenetration of the opposite layers by displacement of the molecules along the *c* axis has been proposed as the most likely transition mechanism.^27,28^

The association of such a displacement with the identified *B*_*g*_ effective lattice vibration is thus straightforward, with a motion that appears to overcome the restoring force in the process that mixes the adjacent layers while maintaining the molecular density. Following this, the monolayer structure typical of the SmE phase can be thought of as resulting from condensation of the soft mode eigenvectors (Figure 6b). Notice that instead the softening of the total-symmetric *A*_*g*_ counterpart of the vibrational motion would produce the collapse of two layers into one, also changing the macroscopic dimensions of the sample and contradicting the experimental observations.

In the mode softening stage, the lattice phonon spectra display a seamless evolution in temperature. However, it is the discontinuity detected in the in-plane *bb* and *ab* spectra at the onset of the transition, i.e., above 418 K, which identifies the actual lattice transformation (see Figure 5, left panel). In fact, the sudden band broadening indicates an in-plane rearrangement that follows the layer mixing. This is consistent with the BTBT cores assuming a new herringbone structure in the SmE phase,^29^ due to the CH−π interactions, which are established by rotation around the long molecular axes ultimately resulting in a monolayer rather than bilayer arrangement.

## Conclusions

By carrying out the study on single crystals, rather than on polycrystalline samples or thin films, the spectral features of the Crystal to SmE transformation of Ph-BTBT-10 could be related to the lattice dynamics along specific crystal directions and therefore to the anisotropic properties of the system.

The two step mechanism revealed by the spectroscopic approach involves first the interpenetration of the molecular layers of the crystal driven by an effective soft mode, followed by the discontinuous intralayer rearrangement of the molecule rigid cores into the herringbone motif of the final phase. The former process in fact anticipates the transition, and the softening entails lattice vibrations with a translational component along the layer shifting direction. The latter displays instead the fingerprint of discontinuity in the abrupt spectral changes at the transition, with features typical of crystal to liquid crystal transitions.^25,30−32^

The findings are consistent with the results found in previous works on Ph-BTBT-10. XRD measurements on oriented thin films demonstrated that the layers maintain the same orientation through the Crystal to SmE transition, while a herringbone packing still characterizes the ordered SmE phase.^16^ An interlayer translation of the molecules as a first step of the transformation was also proposed by Molecular Dynamics simulations.^27,28^

These observations show the predominant displacive character of the transition with the key role played by cooperative lattice vibrations in which the restoring force appears to decay, driving the transformation from bilayer to monolayer. Such a mechanism also explains the effectiveness of thermal treatment of the films in recovering the crystal phase in the reverse transformation. In fact, at the thermal annealing temperature, the crystal is thermodynamically stable, while the vibration involved is sufficiently soft to facilitate the sliding process of the layers.

## Experimental Section

Ph-BTBT-10 was synthesized following the previously reported procedure.^15^ Single crystals were obtained by recrystallization of the synthesized powder in a 1,2-dichlorobenzene solution, and after slow solvent evaporation, white platelets were obtained.

The Raman spectra were recorded with a Horiba LabRAM HR Evolution Raman microspectrometer equipped with a 633 nm HeNe laser and a set of Bragg filters to reject the Rayleigh radiation. The microspectometer was equipped with a diffraction grating with 1800 grooves per mm and an 800 mm focal length allowing for a maximum spectral resolution of 0.3 cm^–1^ and a lowest accessible frequency of 4 cm^–1^. The crystals have sheetlike morphology (typical size 100 × 200 × 5 μm^3^) and tend to overlap. Thus, single crystal domains were selected by microscopic observation using Polarized Optical Microscopy (POM). The measurements were performed in backscattering geometry on both the *bc* and *ab* planes. The experimental setup is described in Figure S1. Since the extended face is parallel to the *ab* plane, the measurements on the *bc* plane required a homemade sample holder composed of thin glass slides. A crystal was oriented and fixed between them.

The temperature was controlled in the range 83–430 K using a Linkam HFS 91 stage, fitted under the microscope. When comparing spectra recorded at different temperatures, the raw data were converted into the imaginary part of the dynamic susceptibility χ*″*), as described in refs (25) and (33). This corrects the intensity enhancement at small wavenumbers due to the thermal excitation of vibrational modes according to the following relationships:
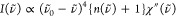

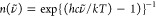
here *I*(ν̃) is the recorded intensity, *χ″*(ν̃) is the imaginary component of the susceptibility, ν̃_0_ is the wavenumber of the exciting light, ν̃ is the Raman shift in wavenumber, and *n*(ν̃) is the Bose–Einstein factor.

## Supporting Information Available

Experimental configuration and crystal orientation of the polarized Raman measurements; BFDH morphology of the Ph-BTBT-10 crystal; polarized low frequency Raman spectra of a unsubstituted BTBT single crystal; and temperature dependent spectra (PDF)

## References

[ref1] Fleury P. A.; Scott J. F.; Worlock J. M. Soft Phonon Modes and the 110° K Phase Transition in SrTiO3. Phys. Rev. Lett. 1968, 21, 16–19. 10.1103/PhysRevLett.21.16.

[ref2] Cowley R. A.; Buyers W. J. L.; Dolling G. Relationship of normal modes of vibration of strontium titanate and its antiferroelectric phase transition at 110° K. Solid State Commun. 1969, 7, 181–184. 10.1016/0038-1098(69)90720-0.

[ref3] Cochran W. Soft modes, a personal perspective. Ferroelectrics 1981, 35, 3–8. 10.1080/00150198108017658.

[ref4] Dove M. T. Theory of displacive phase transitions in minerals. Am. Mineral. 1997, 82, 213–244. 10.2138/am-1997-3-401.

[ref5] Masino M.; Girlando A.; Soos Z. G. Evidence for a soft mode in the temperature induced neutral-ionic transition of TTF-CA. Chem. Phys. Lett. 2003, 369, 428–433. 10.1016/S0009-2614(03)00002-2.

[ref6] Dressel M.; Peterseim T. Infrared Investigations of the Neutral-Ionic Phase Transition in TTF-CA and Its Dynamics. Crystals 2017, 7, 17 10.3390/cryst7010017.

[ref7] Ferrari E.; Mezzadri F.; Masino M. Temperature-induced neutral-to-ionic phase transition of the charge-transfer crystal tetrathiafulvalene-fluoranil. Phys. Rev. B 2022, 105, 054106 10.1103/PhysRevB.105.054106.

[ref8] Anwar J.; Tuble S. C.; Kendrick J. Concerted Molecular Displacements in a Thermally-Induced Solid-State Transformation in Crystals of DL-Norleucine. J. Am. Chem. Soc. 2007, 129, 2542–2547. 10.1021/ja066686y. 17286403

[ref9] Iino H.; Hanna J.-i. Liquid crystalline thin films as a precursor for polycrystalline thin films aimed at field effect transistors. J. Appl. Phys. 2011, 109, 074505 10.1063/1.3563586.

[ref10] Iino H.; Kobori T.; Hanna J.-i. Improved thermal stability in organic FET fabricated with a soluble BTBT derivative. Journal of non-crystalline solids 2012, 358, 2516–2519. 10.1016/j.jnoncrysol.2012.03.021.

[ref11] Yamashita Y. Organic semiconductors for organic field-effect transistors. Sci. Technol. Adv. Mater. 2009, 10, 024313 10.1088/1468-6996/10/2/024313. 27877286 PMC5090443

[ref12] Koch N. Organic Electronic Devices and Their Functional Interfaces. ChemPhysChem 2007, 8, 1438–1455. 10.1002/cphc.200700177. 17539032

[ref13] Hasegawa T.; Takeya J. Organic field-effect transistors using single crystals. Sci. Technol. Adv. Mater. 2009, 10, 024314 10.1088/1468-6996/10/2/024314. 27877287 PMC5090444

[ref14] Minemawari H.; Tsutsumi J.; Inoue S.; Yamada T.; Kumai R.; Hasegawa T. Crystal structure of asymmetric organic semiconductor 7-Decyl-2-phenyl[1] benzothieno[3,2 – *b*][1] benzothiophene. Applied Physics Express 2014, 7, 091601 10.7567/APEX.7.091601.

[ref15] Iino H.; Usui T.; Hanna J.-i. Liquid crystals for organic thin-film transistors. Nat. Commun. 2015, 6, 6828 10.1038/ncomms7828. 25857435 PMC4403349

[ref16] Hofer S.; Bodlos W.; Novák J.; Sanzone A.; Beverina L.; Resel R. Molecular packing analysis of the crystal smectic E phase of a benzothieno-benzothiophene derivative by a combined experimental/computational approach. Liq. Cryst. 2021, 48, 1888–1896. 10.1080/02678292.2021.1907626.

[ref17] Iino H.; Hanna J.-I. Liquid crystal and crystal structures of a phenyl-benzothienobenzothiophene derivative. Mol. Cryst. Liq. Cryst. 2017, 647, 37–43. 10.1080/15421406.2017.1289427.

[ref18] Hofer S.; Unterkofler J.; Kaltenegger M.; Schweicher G.; Ruzié C.; Tamayo A.; Salzillo T.; Mas-Torrent M.; Sanzone A.; Beverina L.; Geerts Y. H.; Resel R. Molecular Disorder in Crystalline Thin Films of an Asymmetric BTBT Derivative. Chem. Mater. 2021, 33, 1455–1461. 10.1021/acs.chemmater.0c04725. 33642680 PMC7905871

[ref19] Tamayo A.; Hofer S.; Salzillo T.; Ruzié C.; Schweicher G.; Resel R.; Mas-Torrent M. Mobility anisotropy in the herringbone structure of asymmetric Ph-BTBT-10 in solution sheared thin film transistors. J. Mater. Chem. C 2021, 9, 7186–7193. 10.1039/D1TC01288F. PMC8191576 34211720

[ref20] Wu H.; Iino H.; Hanna J. Scalable Ultrahigh-Speed Fabrication of Uniform Polycrystalline Thin Films for Organic Transistors. ACS Appl. Mater. Interfaces 2020, 12, 29497–29504. 10.1021/acsami.0c05105. 32436375

[ref21] Donnay J. D. H.; Harker D. A new law of crystal morphology extending the Law of Bravais. Am. Mineral. 1937, 22, 446–467.

[ref22] Hartshorne N. H.; Stuart A. Crystals and the polarising microscope: a handbook for chemists and others; Edward Arnold Ltd.: 1960.

[ref23] Bedoya-Martínez N.; Schrode B.; Jones A. O. F.; Salzillo T.; Ruzié C.; Demitri N.; Geerts Y. H.; Venuti E.; Della Valle R. G.; Zojer E.; Resel R. DFT-Assisted Polymorph Identification from Lattice Raman Fingerprinting. J. Phys. Chem. Lett. 2017, 8, 3690–3695. 10.1021/acs.jpclett.7b01634. 28731723 PMC5545759

[ref24] Schweicher G.; d’Avino G.; Ruggiero M. T.; Harkin D. J.; Broch K.; Venkateshvaran D.; Liu G.; Richard A.; Ruzié C.; Armstrong J.; et al. Chasing the “killer” phonon mode for the rational design of low-disorder, high-mobility molecular semiconductors. Adv. Mater. 2019, 31, 1902407 10.1002/adma.201902407. 31512304

[ref25] Nakayama H.; Minagawa Y.; Abematsu C.; Yajima S.; Ishii K. Pseudo-lattice vibrations in smectic phase of liquid crystals: studies on small wave number Raman spectra of 4-alkyl-4’-cyanobiphenyl. Chem. Phys. 2000, 253, 331–337. 10.1016/S0301-0104(99)00397-3.

[ref26] Hyun B.-R.; Quitevis E. L. Low-frequency spectrum of homeotropically aligned liquid crystals: optical heterodyne-detected Raman-induced Kerr effect spectroscopy of 4-octyl-4’-cyanobiphenyl. Chem. Phys. Lett. 2003, 370, 725–732. 10.1016/S0009-2614(03)00205-7.

[ref27] Baggioli A.; Casalegno M.; Raos G.; Muccioli L.; Orlandi S.; Zannoni C. Atomistic Simulation of Phase Transitions and Charge Mobility for the Organic Semiconductor Ph-BTBT-C10. Chem. Mater. 2019, 31, 7092–7103. 10.1021/acs.chemmater.9b02882.

[ref28] Yoneya M. Monolayer Crystal Structure of the Organic Semiconductor 7-Decyl-2-phenyl[1] benzothieno[3,2 – *b*][1] benzothiophene. J. Phys. Chem. C 2018, 122, 22225–22231. 10.1021/acs.jpcc.8b04386.

[ref29] Saito K.; Miyazawa T.; Fujiwara A.; Hishida M.; Saitoh H.; Massalska-Arodź M.; Yamamura Y. Reassessment of structure of smectic phases: Nano-segregation in smectic E phase in 4-n-alkyl-4’-isothiocyanato-1, 1’-biphenyls. J. Chem. Phys. 2013, 139, 114902 10.1063/1.4821162. 24070306

[ref30] Borer W.; Mitra S.; Brown C. Crystal to liquid-crystal transition studied by Raman scattering. Phys. Rev. Lett. 1971, 27, 379 10.1103/PhysRevLett.27.379.

[ref31] Bulkin B. J.; Prochaska F. T. Vibrational spectra of liquid crystals. II. The Raman spectrum of p-azoxyanisole in crystal, nematic, and isotropic phases, 10–100^–1^ region. J. Chem. Phys. 1971, 54, 635–639. 10.1063/1.1674888.

[ref32] Nakayama H.; Minagawa Y.; Yajima S.; Hyuga T.; Ishii K. Pseudo-lattice vibration in smectic liquid crystals. Physica B: Condensed Matter 1999, 263, 835–838. 10.1016/S0921-4526(98)01466-5.

[ref33] Nakayama H.; Yajima S.; Yoshida T.; Ishii K. Relaxational Molecular Motions in Simple Organic Liquids: Studies with Low-Wavenumber Depolarized Raman Spectroscopy. J. Raman Spectrosc. 1997, 28, 15–22. 10.1002/(SICI)1097-4555(199701)28:1<15::AID-JRS56>3.0.CO;2-W.

