# Supplementary material for: Interlayer Sliding Phonon Drives Phase Transition in the Ph-BTBT-10 Organic Semiconductor
Source: Chem Mater. 2023 Jul 20;35(15):5777–83. doi: 10.1021/acs.chemmater.3c00209 (PMC10413852; doi:10.1021/acs.chemmater.3c00209)
Supplement: Supplementary file 1 — cm3c00209_si_001.pdf [file cm3c00209_si_001.pdf]

Interlayer sliding phonon drives phase transition in Ph-BTBT-10 organic semiconductor

Elena Ferrari,† Lorenzo Pandolfi,‡ Guillaume Schweicher,¶ Yves Geerts,¶,§ Tommaso Salzillo,‡ Matteo Masino,*,† and Elisabetta Venuti*,‡

†*Dipartimento di Scienze Chimiche, della Vita e della Sostenibilità Ambientale & INSTM-UdR Parma, Parco Area delle Scienze, 17/A, 43124, Parma, Italy*

‡*Dipartimento di Chimica Industriale "Toso Montanari" & INSTM-UdR Bologna, Viale del Risorgimento 4 - 40136, Bologna, Italy*

¶*Laboratoire de Chimie des Polymères Faculté des Sciences Université Libre de Bruxelles (ULB), Boulevard du Triomphe, CP 206/01, Brussels 1050, Belgium*

§*International Solvay Institutes of Physics and Chemistry Université Libre de Bruxelles (ULB), Boulevard du Triomphe, CP 206/01, Brussels 1050, Belgium*

E-mail: matteo.masino@unipr.it; elisabetta.venuti@unibo.it

Experimental details

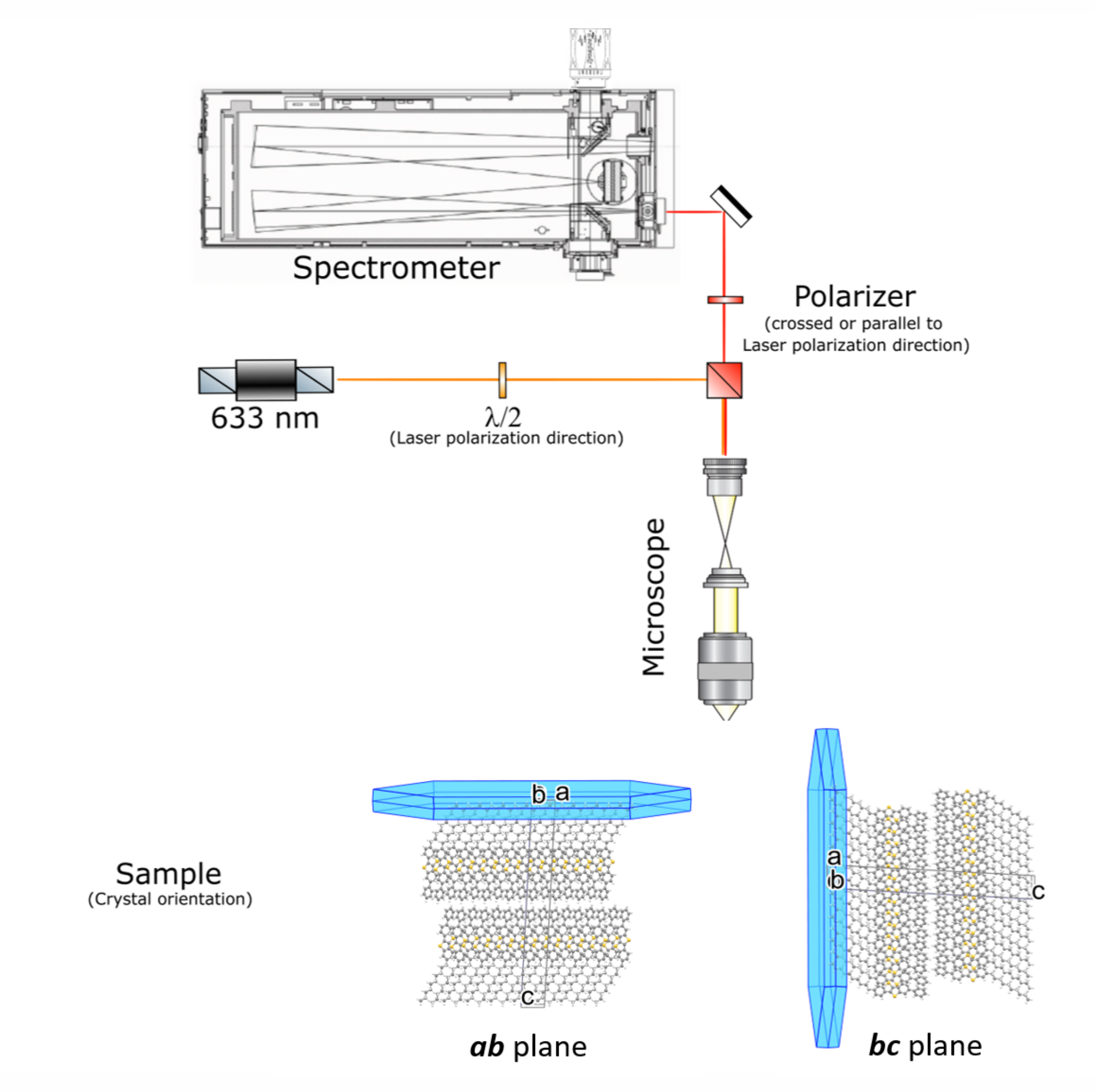

Figure S1: Experimental configuration and crystal orientation of the polarized Raman measurements.

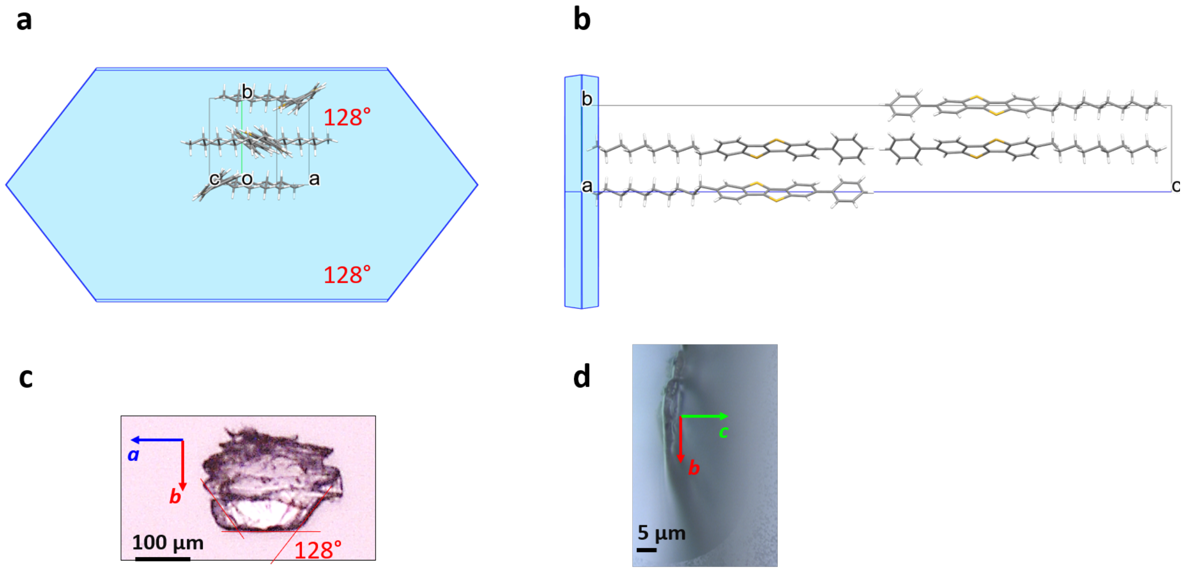

Figure S2: BFDH morphology of Ph-BTBT-10 crystal, viewed along a) c* and b) a* directions together with the unit cell. The microscopic images of a crystal viewed along the same directions are shown in c) and d).

Comparison with unsubstituted BTBT

Similar to Ph-BTBT-10, BTBT crystallizes in a layered structure with an in-plane herringbone arrangement of the molecules (CCDC refcode PODKEA, space group $P2_1/c$). While Ph-BTBT-10 unit cell contains Z=4 molecules per cell in general position, BTBT has Z=2 with the two molecules located on the inversion centers. Thus, in BTBT the only active Raman phonons are the 6 librations of gerade symmetry, being the translations all of underage symmetry and not active.

The low frequency mode found at 45 cm^{-1} , has the same intensity in the in-plane and out-of-plane polarized spectra, while the higher frequency modes are completely in-plane polarized. According to the calculations reported in Phys. Chem. Chem. Phys., 23, 15485-15491 (2021), the latter vibrations correspond to rotations about the axis perpendicular to the plane and about the long axis of the molecule. Thus, the Ph-BTBT-10 low-frequency phonons, completely interlayer polarized, can be safely assigned to translations along the long axis or wagging of the decyl chains. Furthermore, the in-plane polarized modes at 90-100 cm^{-1} are assigned to rotations around the long axes.

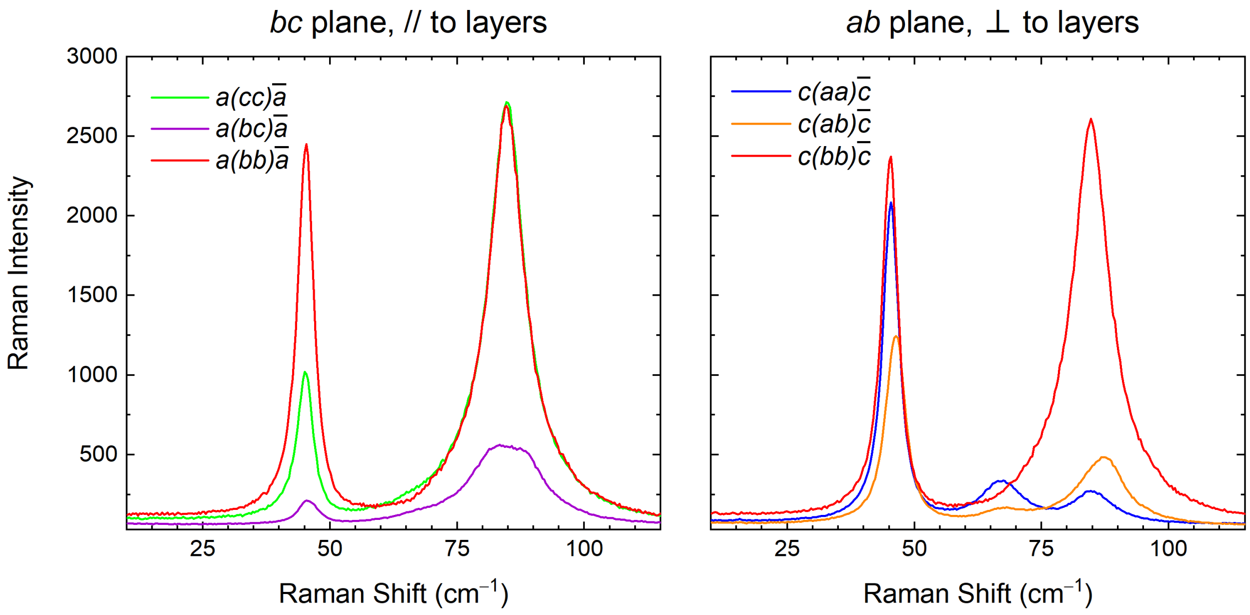

Figure S3: Polarized low frequency Raman spectra of a BTBT single crystal, measured on the *bc* and *ac* planes.

Temperature dependent spectra

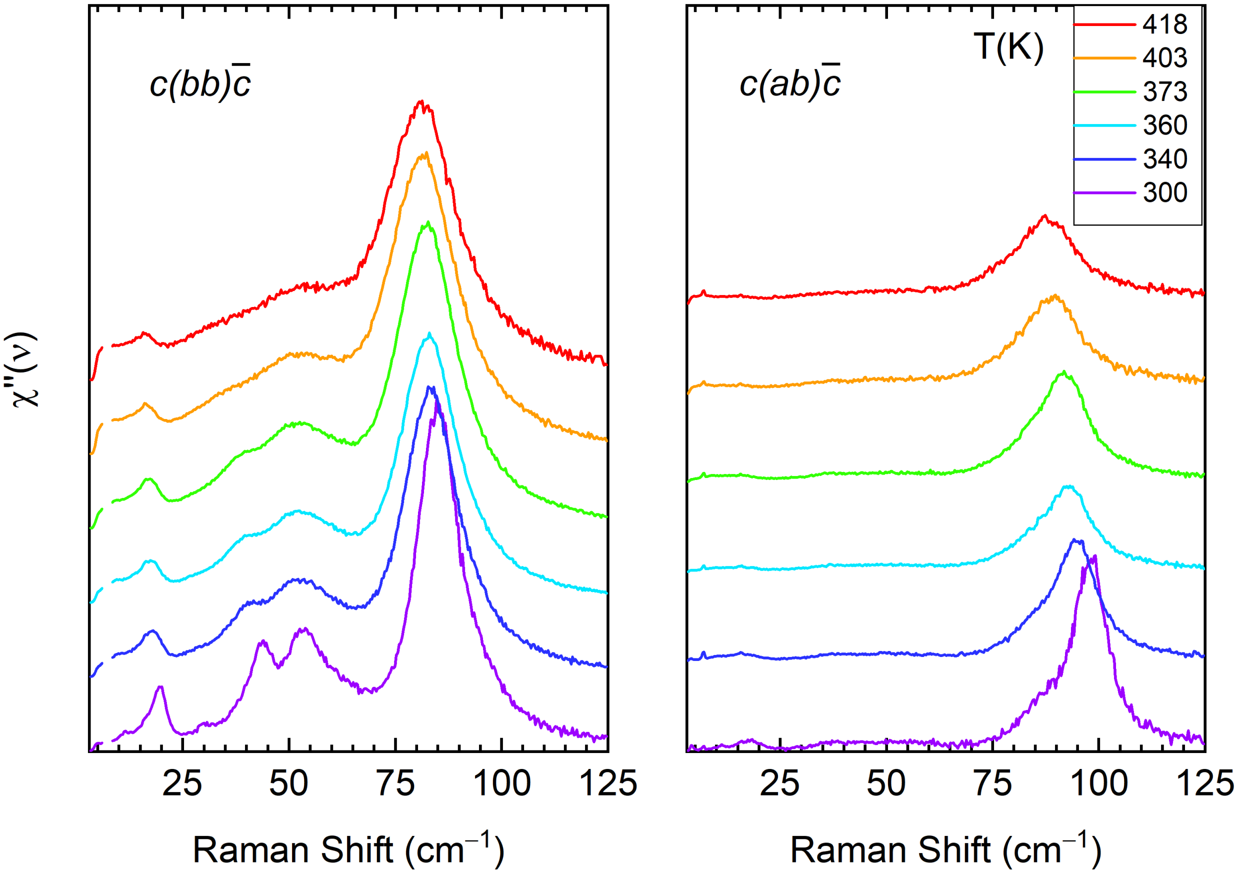

Figure S4: Temperature dependent *bb* and *ab* polarized spectra

The temperature dependent frequencies of the *bc* and *cc* polarized phonons, shown in Fig S5, were obtained by fitting the low wavenumber region (4-30 cm^{-1}) of the corresponding spectra. Three Lorentzian functions have been used to fit the three lowest frequency modes. Above 340 K two Lorentzian functions were used instead, excluding the 4-7 cm^{-1} range, as the band at 4 cm^{-1} was no longer accessible. The 84 K spectra were fitted firstly and the obtained parameters were used as input for the next temperature spectrum. The R-Square was always > 0.95.

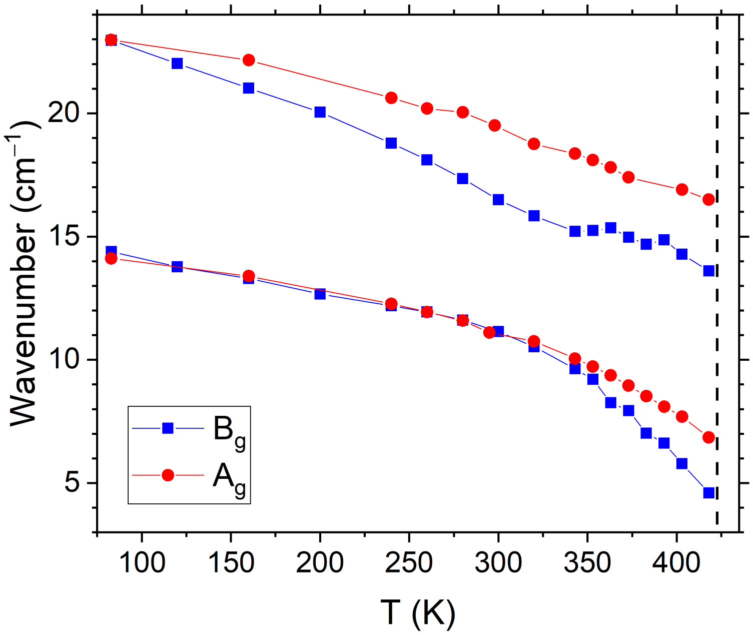

Figure S5: Temperature dependent frequency shifts of the bands detected at 14 and 23 cm^{-1} at 83 K in the two polarizations analyzed in this work (see main text). The A_g and B_g modes initially superimposed, behave differently on increasing temperature.
